# Supplementary material for: Direct electrical stimulation of the premotor cortex shuts down awareness of voluntary actions
Source: Nat Commun. 2020 Feb 4;11:705. doi: 10.1038/s41467-020-14517-4 (PMC7000749; doi:10.1038/s41467-020-14517-4)
Supplement: Supplementary file 1 — Description of Additional Supplementary Files [file 41467_2020_14517_MOESM1_ESM.pdf]

Description of Additional Supplementary Files

File Name: Supplementary Movie 1

Description: The video recording shows the effect of DES applied over premotor cortex (PMC) while the patients were performing the HMt coupled with MMt. The patients neither were aware of the exact timing of the stimulation nor could monitor the hand movement by sight. The video recording of the hand during HMt is reproduced in the upper left panel. A lightning represents the time in which the stimulation was delivered. The video recording of the surgical field is shown in the upper right panel. Medial, anterior, posterior, and lateral reference frames indicate the surgical field orientation. DES was delivered by a bipolar probe. The lighting and the artificial sound of the DES were synchronized on the onset and offset of the stimulation of the PMC (Supplementary Video 1). In compliance with the Ethical Regulation, the patient's audio file of the voice during MMt cannot be recorded during intraoperative procedure, thus the audio of an actor reproducing the patient's voice performing the MMt was used. In the bottom panel, the digitalized trace of the patient's voice is shown. Despite DES applied over PMC induced a significant impairment of the hand movement, the patient was not aware of the motor arrest and, indeed, kept saying "OK" as if the HMt was correctly performed.

File Name: Supplementary Movie 2

Description: The video recording shows the effect of DES applied over the primary somatosensory cortex (S1) while the patients were performing the HMt coupled with MMt. The patients neither were aware of the exact timing of the stimulation nor could monitor the hand movement by sight. The video recording of the hand during HMt is reproduced in the upper left panel. A lightning represents the time in which the stimulation was delivered. The video recording of the surgical field is shown in the upper right panel. Medial, anterior, posterior, and lateral reference frames indicate the surgical field orientation. DES was delivered by a bipolar probe. The lighting and the artificial sound of the DES were synchronized on the onset and offset of the stimulation of S1. In compliance with the Ethical Regulation, the patient's audio file of the voice during MMt cannot be recorded during intraoperative procedure, thus the audio of an actor reproducing the patient's voice performing the MMt was used. In the bottom panel, the digitalized trace of the patient's voice is shown. DES applied over S1 produced a significant impairment of the hand movement (comparable to that induced by the PMC stimulation), but the patient was fully aware of it and, indeed, she reported "STOP" during the movement arrest.
